# Distinct macular thickness changes after femtosecond laser–assisted cataract surgery of age-related cataract and myopia with cataract

**DOI:** 10.1038/s41598-018-21698-y

**Published:** 2018-02-19

**Authors:** Yong Wang, Jun Du, Mei Yang, Yi Xu, Huaijin Guan, Jian Wu

**Affiliations:** Eye Institute, Affiliated Hospital of Nantong University, Nantong, Jiangsu China

## Abstract

Cataract surgery can cause macular thickness change. We used optical coherence tomography (OCT) to assess the macular thickness of different regions after femtosecond laser–assisted cataract surgery of age-related cataracts (ARC) and myopia cases with cataract (myopia group). Fifty eyes of 50 patients in ARC group and fifty eyes of 50 patients in myopia with cataract group were included. All study underwent femtosecond laser–assisted cataract surgery and macular thickness of was measured at pre-operation and 1 week, 1 month after surgery. There are significant differences of foveal thickness (*P* = 0.02), foveal volume (*P* = 0.02) and average retinal thickness (*P* = 0.02) between two groups before operation. In the myopia group, statistically significant differences were not found in postoperative macular thickness as compared with ARC group. There are differences in macular thickness between pre-operation and 1 month after operation when compared with nasal outer macular ring thickness (*P* = 0.022), foveal volume (*P* = 0.005) and average retinal thickness (*P* = 0.012) in ARC group. The study suggest that femtosecond laser–assisted cataract extraction is safe in myopia group that did not cause significant increase of macular thickness. However, an increased post-operative local macular thickness was recoded while comparing macular thickness with the baseline in ARC group.

## Introduction

The subclinical macular edema after common and complex cataract surgery has brought substantial attention to surgeons due to the potential hazard of this complication to vision consequence^1,2^. Until now, the etiology and pathogenesis for the complication have not been completely clarified. Studies have showed that the anterior segment inflammation may play a vital role in the pathology after cataract surgery^3,4^. Other researches also showed that the retina pulled by posterior vitreous might contribute to the etiologic mechanism. It has been suggested that the phenomena can be caused by the shock wave of the phacoemulsification during cataract surgery. In myopia, complete or incomplete posterior vitreous detachment (PVD) is common phenomena due to fluidity of vitreous, which also presents after cataract surgery. During the process, macular pulled by vitreous may play a vital role for formation of subclinical macular edema. These findings support the notion that myopic eyes are more prone to macular edema formation after cataract surgery. But it is warranted for further study whether it is the case in femtosecond laser-assisted cataract surgery.

Femtosecond laser technology has become widely applied in various ophthalmic surgery especially for cataract^5^. On one hand, it brings precise surgical incision. On the other hand, it reduces the phacoemulsification power and improves the outcomes of cataract surgery. However, some surgeons have concerns on the suction ring used during the femtosecond laser procedure to avoid eye movements due to vacuum aspiration. A report showed that the suction ring causes temporally increase of intraocular pressure (IOP) during the vacuum aspiration (up to 40 mmHg in the LenSx laser system, Alcon Lasers, Inc.)^6^, which can induce various changes from anterior segment to posterior segment of ocular structure^7^. There is an increase of the vitreous distance using microkeratome suction ring for LASIK^8^. These alterations can cause a series of changes including bilateral vitreous detachment^9^, retinal hemorrhage^10^ and the change of ocular blood-flow^11^.

Optical coherence tomography (OCT) has been widely used in ophthalmic applications which is a noninvasive and high-resolution imaging modality for ocular internal structures^12^. Several studies used OCT to detect an increase of the parafoveal retinal thickness, foveal volume, and volume of the entire macula in glaucomatous eyes, manual small incision cataract surgery and phacoemulsification cataract surgery^13–15^. Studies also used OCT to assess the macular morphology after laser–assisted cataract surgery^1,16^. However, no study focuses on and compares the change of macular region thickness between ARC (age-related cataract) and myopic with cataract after femtosecond laser–assisted cataract surgery. The information of retinal thickness changes after the cataract operation especially for myopic eyes can provide insight to pathophysiology of subclinical macular edema occurring.

## Patients and Methods

### Patients

The study included 50 eyes from 50 patients with ARC and 50 eyes from 50 myopic with cataract (myopia group). The axial length is 23.03 ± 0.67 mm in the ARC group and 29.15 ± 4.17 mm in the myopia group. We excluded the patients with previous ocular surgery, trauma and known macular alteration (diabetic retinopathy or age-related macular degeneration). All patients were given a complete ophthalmologic evaluation before surgery including refraction, a slit lamp examination, B-scan ultrasonography and a dilated fundus evaluation.

The study received approval of the ethics committee at Affiliated Hospital of Nantong University and was conducted in compliance with the Declaration of Helsinki. All patients were willing to volunteer for the research and signed a written informed consent.

### Surgery

All surgeries were performed by the same surgeon (H.J.G.) using the CENTURION^®^ VISION SYSTEM (Alcon Laboratories Inc, Ft Worth, Texas). After pupillary dilation and topical anesthetics, a curved contact lens was used to applanate the cornea by the LenSx laser system. OCT was used to detect the location of the crystalline lens surface. After scanning a cylindrical pattern, a 5.5-mm diameter capsulotomy procedure was performed starting at 100 µm below the anterior capsule and ending at 100 µm above the capsule. The lens was fragmented into eight quadrants by a cross pattern. A self-sealing biplanar corneal incision (2.4 mm) and a side-port incision (1.0 mm) were created by femtosecond laser. In the study, proprietary energy and spot separation parameters used were 10 µJ spot and layer separation 8 µm for lens fragmentation, 6 µJ for capsulotomy, and 6-µJ spot separation 6 µm and layer separation 3 µm for primary and secondary corneal incision.

After treatment by femtolaser, the self-sealing corneal incisions was made by a blunt spatula and viscoelastic material was injected to the anterior chamber. A 5.5-mm diameter capsulotomy outside anterior chamber was identified and removed by rhexis forceps, followed by hydrodissection. The lens was removed using a stop-and-chop or a divide-and-conquer technique. The cortex was completely removed and then a one-piece, hydrophobic, acrylic, posterior chamber lens (SN60WF; Alcon Laboratories, Inc.; Fort Worth, TX) was implanted. At last, the viscoelastic material was removed by irrigation-aspiration. No any intra- or postoperative complications occurred during the surgery in the study.

### Oct Measurements

OCT measurements (Cirrus HD-OCT 4000; Carl Zeiss Meditec, Dublin, CA) were performed 1 day before surgery and post-operation at 1 week and 1 month. The selection of the time points was based on that the most retinal thickening is observable after conventional phacoemulsification at those time points. The different macular region thickness was automatically determined by the instrument software as the distance between the internal limiting membrane and retinal pigment epithelium. Measurements were provided for three concentric regions. The central foveal region was a region with a diameter of 1 mm, and the inner and outer rings had outer diameters of 3 and 6 mm, respectively, and were divided into four quadrants respectively. We described them as superior outer macular ring thickness (SOMRT), bitamporal outer macular ring thickness (BOMRT), inferior outer macular ring thickness (IOMRT), nasal outer macular ring thickness (NOMRT), superior inter macular ring thickness (SIMRT), bitamporal inter macular ring thickness (BIMRT), inferior inter macular ring thickness (IIMRT), nasal inter macular ring thickness (NIMRT), foveal thickness (FT), foveal volume (FV) and average retinal thickness (ART).

### Statistical Analysis

A SPSS 18.0 software (SPSS Inc, Chicago, Illinois) was performed for statistical analyses. Data are expressed as the mean and standard deviation. For comparisons of different macular region thickness between pre-operation and post-operation of femtosecond laser–assisted cataract surgery, the student’s t test was used. Pearson’s correlation analysis was used to assess the correlation between the nuclear hardness, phaco time, cumulative dissipated energy (CDE) and different macular region thickness. A *P* value less than 0.05 was considered statistically significant.

## Results

### Patient Characteristics

The ARC group comprised of 50 eyes of 50 patients with the mean age of 61.33 ± 7.52 years (range 49 to 76 years). The myopia group comprised of 50 eyes of 50 patients with the mean age of 56.66 ± 5.68 years (range 46 to 63 years) (Table 1).

**Table 1:** Comparison of ARC Group and Myopia Group.

Demographic	Myopia Group	ARC Group	*P* Value
Age (y)	56.66 ± 5.68	61.33 ± 7.52	0.14
phaco time (s)	13.62 ± 11.64	30.87 ± 22.72	0.52
CDE	8.76 ± 7.93	12.37 ± 8.7	0.34
nuclear hardness	2.11 ± 0.33	2.42 ± 0.51	0.15

CDE = cumulative dissipated energy.

### Oct Parameters

Table 2 shows the pre- and postoperative macular thickness values in the ARC and myopia groups. The macular thicknesses measured in FT, FV and ART in myopia group are significantly higher than the ARC group in the baseline measurement (pre-operative). There are not statistically significant after surgery between the two groups. There are not statistically significant after surgery between the two groups. But in ARC group, we found there are different between pre-operation and post-operation in NOMRT, FV and ART (Tables 2 and 3). However, we did not detect any correlations between the nuclear hardness, phaco time, cumulative dissipated energy (CDE) and the macula thickness (Table 4).

**Table 2:** Retinal Thickness Values After Femtosecond Laser–assisted Phacoemulsification.

Time/Parameter	Mean ± SD		*P* Value
	Myopia Group	ARC Group	
Preoperative			
SOMRT	255.44 ± 40.05	266.5 ± 36.72	0.52
BOMRT	237 ± 45.77	271.08 ± 22.29	0.06
IOMRT	243.11 ± 50.29	262.58 ± 13.71	0.29
NOMRT	280.22 ± 27.23	274.75 ± 30.08	0.67
SIMRT	301.67 ± 59.57	299.08 ± 37.82	0.91
BIMRT	259.89 ± 74.15	308.42 ± 17.35	0.09
IIMRT	279.78 ± 68.48	305.83 ± 31.07	0.25
NIMRT	285.44 ± 61.58	310.97 ± 21.62	0.26
FT	206.89 ± 48.05	251.17 ± 28.4	0.02^*^
FV	8.32 ± 1.48	9.56 ± 0.66	0.02^*^
ART	231 ± 41.2	264.83 ± 18.71	0.02^*^
Postoperative			
1 week			
SOMRT	254.56 ± 58.52	284.25 ± 14.76	0.17
BOMRT	248.89 ± 38.19	266.08 ± 9.7	0.22
IOMRT	243.44 ± 37.45	267.67 ± 15.79	0.1
NOMRT	285.22 ± 35.01	293.92 ± 16.37	0.51
SIMRT	299.44 ± 40.20	316.58 ± 21.59	0.22
BIMRT	285 ± 37.08	305.75 ± 16.35	0.15
IIMRT	302.44 ± 34.5	312.83 ± 17.08	0.42
NIMRT	301.66 ± 42.93	318.16 ± 16.22	0.3
FT	224.11 ± 32.98	246.75 ± 22.58	0.08
FV	8.94 ± 1.67	9.99 ± 0.41	0.1
ART	249 ± 46.21	277.08 ± 11.7	0.11
1 month			
SOMRT	264.67 ± 46.63	289.67 ± 29.26	0.18
BOMRT	245.44 ± 38.99	266.83 ± 22.55	0.13
IOMRT	247 ± 43.5	273.92 ± 17.98	0.11
NOMRT	290 ± 32.7	306.5 ± 31.28	0.26
SIMRT	311.11 ± 41.73	330.92 ± 37.01	0.27
BIMRT	294.78 ± 43.22	316.42 ± 33.31	0.23
IIMRT	310.78 ± 35.95	327.83 ± 39.74	0.32
NIMRT	307.78 ± 46.1	330.25 ± 38.66	0.24
FT	234.44 ± 37.59	257.58 ± 40.91	0.2
FV	9.18 ± 1.81	10.34 ± 0.52	0.09
ART	255.22 ± 49.79	285.67 ± 16.95	0.11

SOMRT = superior outer macular ring thickness; BOMRT = bitamporal outer macular ring thickness; IOMRT = inferior outer macular ring thickness; NOMRT = nasal outer macular ring thickness; SIMRT = superior inter macular ring thickness; BIMRT = bitamporal inter macular ring thickness; IIMRT = inferior inter macular ring thickness; NIMRT = nasal inter macular ring thickness; FT = foveal thickness; FV = foveal volume; ART = average retinal thickness, *P < 0.05.

**Table 3:** The Time Points for Comparison of Retinal Thickness Values.

Visit	Myopia Group (Mean ± SD)			*p*	ARC Group (Mean ± SD)			*p*
	Preoperative	Postoperative	Postoperative		Preoperative	Postoperative	Postoperative	
		1 week	1 month			1 week	1 month	
SOMRT	255.44 ± 40.05	254.56 ± 58.52	264.67 ± 46.63	0.89	266.5 ± 36.72	284.25 ± 14.76	289.67 ± 29.26	0.129
IOMRT	237 ± 45.77	248.89 ± 38.19	245.44 ± 38.99	0.821	271.08 ± 22.29	266.08 ± 9.7	266.83 ± 22.55	0.789
LOMRT	243.11 ± 50.29	243.44 ± 37.45	247 ± 43.5	0.979	262.58 ± 13.71	267.67 ± 15.79	273.92 ± 17.98	0.233
NOMRT	280.22 ± 27.23	285.22 ± 35.01	290 ± 32.7	0.81	274.75 ± 30.08	293.92 ± 16.37	306.5 ± 31.28	0.022^*^
SIMRT	301.67 ± 59.57	299.44 ± 40.20	311.11 ± 41.73	0.861	299.08 ± 37.82	316.58 ± 21.59	330.92 ± 37.01	0.075
BIMRT	259.89 ± 74.15	285 ± 37.08	294.78 ± 43.22	0.383	308.42 ± 17.35	305.75 ± 16.35	316.42 ± 33.31	0.523
IIMRT	279.78 ± 68.48	302.44 ± 34.5	310.78 ± 35.95	0.394	305.83 ± 31.07	312.83 ± 17.08	327.83 ± 39.74	0.216
NIMRT	285.44 ± 61.58	301.66 ± 42.93	307.78 ± 46.1	0.635	310.97 ± 21.62	318.16 ± 16.22	330.25 ± 38.66	0.229
FT	206.89 ± 48.05	224.11 ± 32.98	234.44 ± 37.59	0.353	251.17 ± 28.4	246.75 ± 22.58	257.58 ± 40.91	0.702
FV	8.32 ± 1.48	8.94 ± 1.67	9.18 ± 1.81	0.537	9.56 ± 0.66	9.99 ± 0.41	10.34 ± 0.52	0.005^*^
ART	231 ± 41.2	249 ± 46.21	255.22 ± 49.79	0.518	264.83 ± 18.71	277.08 ± 11.7	285.67 ± 16.95	0.012^*^

SOMRT = superior outer macular ring thickness; BOMRT = bitamporal outer macular ring thickness; IOMRT = inferior outer macular ring

thickness; NOMRT = nasal outer macular ring thickness; SIMRT = superior inter macular ring thickness; BIMRT = bitamporal inter macular ring thickness; IIMRT = inferior inter macular ring thickness; NIMRT = nasal inter macular ring thickness; FT = foveal thickness; FV = foveal volume; ART = average retinal thickness; CDE = cumulative dissipated energy.

**Table 4:** The Correlation between Nuclear Hardness, Phaco Time, CDE and Macular thickness after Operation (ARC Group).

	postoprative (1 week)											postoprative (1 month)										
	SOMRT	BOMRT	IOMRT	NOMRT	SIMRT	BIMRT	IIMRT	NIMRT	FT	FV	ART	SOMRT	BOMRT	IOMRT	NOMRT	SIMRT	BIMRT	IIMRT	NIMRT	FT	FV	ART
nuclear hardness	−0.44	−0.16	−0.1	−0.56	−0.25	−0.09	0.03	−0.12	−0.1	−0.3	−0.34	−0.57	−0.33	−0.53	−0.54	−0.51	−0.39	−0.5	−0.43	−0.35	−0.5	−0.5
phaco time	0.05	−0.11	0.2	0.27	0.04	−0.2	−0.25	−0.1	0.02	0.28	0.29	0.03	0.17	−0.03	0.16	0.07	−0.11	−0	−0.01	−0.06	0.36	0.22
CDE	−0.48	−0.58	−0.14	−0.32	−0.36	−0.42	−0.3	−0.37	−0.3	−0.4	−0.37	−0.51	−0.33	−0.56	−0.47	−0.46	−0.47	−0.4	−0.43	−0.49	−0.5	−0.5

SOMRT = superior outer macular ring thickness; BOMRT = bitamporal outer macular ring thickness; IOMRT = inferior outer macular ring thickness; NOMRT = nasal outer macular ring thickness; SIMRT = superior inter macular ring thickness; BIMRT = bitamporal inter macular ring thickness; IIMRT = inferior inter macular ring thickness; NIMRT = nasal inter macular ring thickness; FT = foveal thickness; FV = foveal volume; ART = average retinal thickness; CDE = cumulative dissipated energy.

## Discussion

As a new technology, femtosecond laser applications in cataract surgery has several advantages including the incision stability^17^, the accurate IOL centration^18–20^ and the efficient lens fragmentation^21,22^. However, the suction ring used during the positioning of the femtosecond laser may have some harmful effect on macular region especially for the complex cataract cases.

In current clinical study, we analyzed the preoperative and postoperative macular thickness values in the myopia and ARC group by OCT. Postoperative cystoid macular edema (CME) is often asymptomatic and can be detected with only fluorescein angiography or OCT. Our results show that there are significant differences between the two groups when compared with FT, FV and ART before operation (Table 2). The results are consisted with previous research that the thicknesses significantly lower in the high myopic eyes than in the nonmyopic eyes^23^. The elongated axial length may be the reason for thinner macular thickness in myopic eyes^24^.

CME can be detected at the first week up to 6 months and peaks 4 to 6 weeks after surgery^25^. In this study, we did not find any difference between the two groups when compared the macular thickness of different region at 1 week or 1 month after surgery between the two groups. But in ARC group, we found there are different between pre-operation and post-operation in NOMRT, FV and ART (Table 3). However, we did not detect any correlations the nuclear hardness, phaco time, CDE and the macula thickness (Table 4). We speculate that the nuclear hardness and phaco time maybe are a crucial factor for the change. The means of nuclear hardness and phaco time is higher in ARC group when compare with myopia group. Though there are not any statistically significant between the two groups for nuclear hardness and phaco time. We also did not detect any statistically significant thickness change at myopia group after surgery. We speculate the reason is that used femtosecond laser technology reduced the phacoemulsification power and phaco time during surgery. The factors may be the cause for the CME formation.

In this study, the limitation includes a relatively small number patients and short follow-up period. Our original design is follow up at 1 week, 1 month, 3 months and 6 months after surgery. But due to the loss of follow up, we only collected the valid data of 50 patients for each group at 1 week and 1 month in current settings. There are not any different for macular thickness change between the two groups (ARC group: n = 22; Myopia group: n = 23) at 3 months (data not shown). The mean macular thickness of myopia group increased after surgery when compare with pre-operation, but this difference is not statistically significant. Our results suggest that used femtosecond laser applications to extract cataract is equally safe in the myopia group. Femtosecond laser–assisted cataract extraction did not result in macular thickness change at myopia group compared to the ARC group, although it needs to confirm at larger cohorts and longer follow up.

In conclusion, the femtosecond laser might provide us a efficacy surgical technology to treatment the myopic eyes with cataract. It is necessary to conduct the further randomized controlled studies with larger cohorts or other patients particularly with high risk for postoperative CME.
